# Performances of Kevlar and Polyethylene as radiation shielding on-board the International Space Station in high latitude radiation environment

**DOI:** 10.1038/s41598-017-01707-2

**Published:** 2017-05-10

**Authors:** Livio Narici, Marco Casolino, Luca Di Fino, Marianna Larosa, Piergiorgio Picozza, Alessandro Rizzo, Veronica Zaconte

**Affiliations:** 1 Department of Physics, University of Rome Tor Vergata, via della Ricerca Scientifica 1, 00133 Roma, Italy; 2 INFN Roma Tor Vergata, via della Ricerca Scientifica 1, 00133 Roma, Italy

## Abstract

Passive radiation shielding is a mandatory element in the design of an integrated solution to mitigate the effects of radiation during long deep space voyages for human exploration. Understanding and exploiting the characteristics of materials suitable for radiation shielding in space flights is, therefore, of primary importance. We present here the results of the first space-test on Kevlar and Polyethylene radiation shielding capabilities including direct measurements of the background baseline (no shield). Measurements are performed on-board of the International Space Station (Columbus modulus) during the ALTEA-shield ESA sponsored program. For the first time the shielding capability of such materials has been tested in a radiation environment similar to the deep-space one, thanks to the feature of the ALTEA system, which allows to select only high latitude orbital tracts of the International Space Station. Polyethylene is widely used for radiation shielding in space and therefore it is an excellent benchmark material to be used in comparative investigations. In this work we show that Kevlar has radiation shielding performances comparable to the Polyethylene ones, reaching a dose rate reduction of 32 ± 2% and a dose equivalent rate reduction of 55 ± 4% (for a shield of 10 g/cm^2^).

## Introduction

Mitigation of radiation risk is one of the most important issues to be addressed to allow human exploration of deep space^1^. Radiation in a deep space habitat is composed by the Galactic Cosmic Rays (GCR), the radiation associated with solar events, such as the Solar Particle Events (SPEs), and the secondary radiation produced by the interaction of GCR and SPEs with the space habitat hull and/or other intervening material (such as a space suit or an experiment rack). Understanding the radiation shielding features of materials is therefore an important step toward an integrated solution for radiation countermeasures in space, where passive shielding will play a major role.

The goal of such studies is to reduce the radiation risk for the crew to a level As Low As Reasonably Achievable (ALARA), down to the ideal case where the risk for radiation exposure in space is comparable to the one on Earth, not to affect the allowable mission duration. As this goal is still out of reach of the available technology, future mission plans consider ‘additional’ radiation risk, which must lie below some pre-determined threshold.

The study of the radiation shielding performances for a given material to be used in space missions generally follows three main steps. First, shielding capabilities of the material are validated by a Monte Carlo simulation. If results are promising, then the material properties may be evaluated by ions irradiation in particle accelerator facilities. The ions used in these test are expected to be the most abundant ones in space. Finally, a characterization in space are performed only for materials which have shown the best characteristics in the previous two steps.

Measurements in Low Earth Orbit (LEO) at high latitude allow the investigation of a radiation field similar to the deep-space one, with the correct ions spectrum, needed to evaluate the material characteristics as radiation shield. However, these measurements are much more resource demanding than the ground based ones. For this reason, they require a whole preliminary characterization of the material on ground (as mentioned above) and a clear indication that the material is indeed a promising candidate.

The International Space Station (ISS) is the best available laboratory for these tests on material response to space radiation. Even if within the protection of the Earth’s magnetic field, the spectrum of the radiation environment inside the ISS at high latitudes is the closest available replica of the outer space radiation spectrum^2^.

These measurements require an active detection system, which allows to select data acquired only in the appropriate orbital tract (high latitudes). This selection permits the exclusion of the contribution of the South Atlantic Anomaly (SAA) region from the data (the SAA is a region where the Van Allen Belts gets closer to Earth and trap a large amount of low energy protons, generating a radiation field unrelated with the deep-space one).

Radiation flux inside the ISS changes mostly as consequence of i) the modulation of the Galactic Cosmic Rays (GCR) due to solar cycle; ii) the modulation of GCR due to the ISS position within the Earth magnetic field; iii) external events such as solar Coronal Mass Ejections (CMEs) and flares. Moreover, the hull of the ISS and the equipment (often moved inside the ISS, in different sites) behave as a shield for the external radiation with variable density and thickness, and must be considered. To measure shielding effectiveness taking into account such radiation flux variability, concurrent measurements with and without shielding material on the detector are therefore needed.

Monte Carlo simulations and ground tests, as well as a first preliminary test in space, have been performed on Kevlar in the recent past^3–6^. These works show good radiation shielding effectiveness, comparable with the one of Polyethylene. Highly hydrogenated materials perform best as radiation shields in space^1^, since they prevent nuclear fragmentation processes which can enhance the dose. Polyethylene is presently considered as the material that merges a high level of hydrogenation, easiness of handling and machining and affordable cost. It is often taken as a benchmark to compare other materials shielding effectiveness. Kevlar is therefore a very good candidate, considering also its resistance to impacts (important for debris shielding). Moreover, being available as a fabric, it may be easily adapted to other purposes, for example Extra Vehicular Activity (EVA) suits or ‘extra’ shielding in some specific locations of the habitats, such as in the crew sleeping quarters.

In this paper we show the results of the measurements performed in the ISS during the ALTEA-shield project for the investigation of the shielding effectiveness of Polyethylene and Kevlar. Three detectors of the ALTEA system^7^ have been used. These active detectors are able to merge radiation measurements with the ISS position. It is therefore possible to select the measurements in the orbital tract that best mimics the radiation expected in a deep space environment^8^. Moreover, being the three detectors identical, they are suitable to be used concurrently: one for the unshielded baseline and the other two to measure radiation with two different amounts of the same material as shield.

## Results

**Figure 1:**
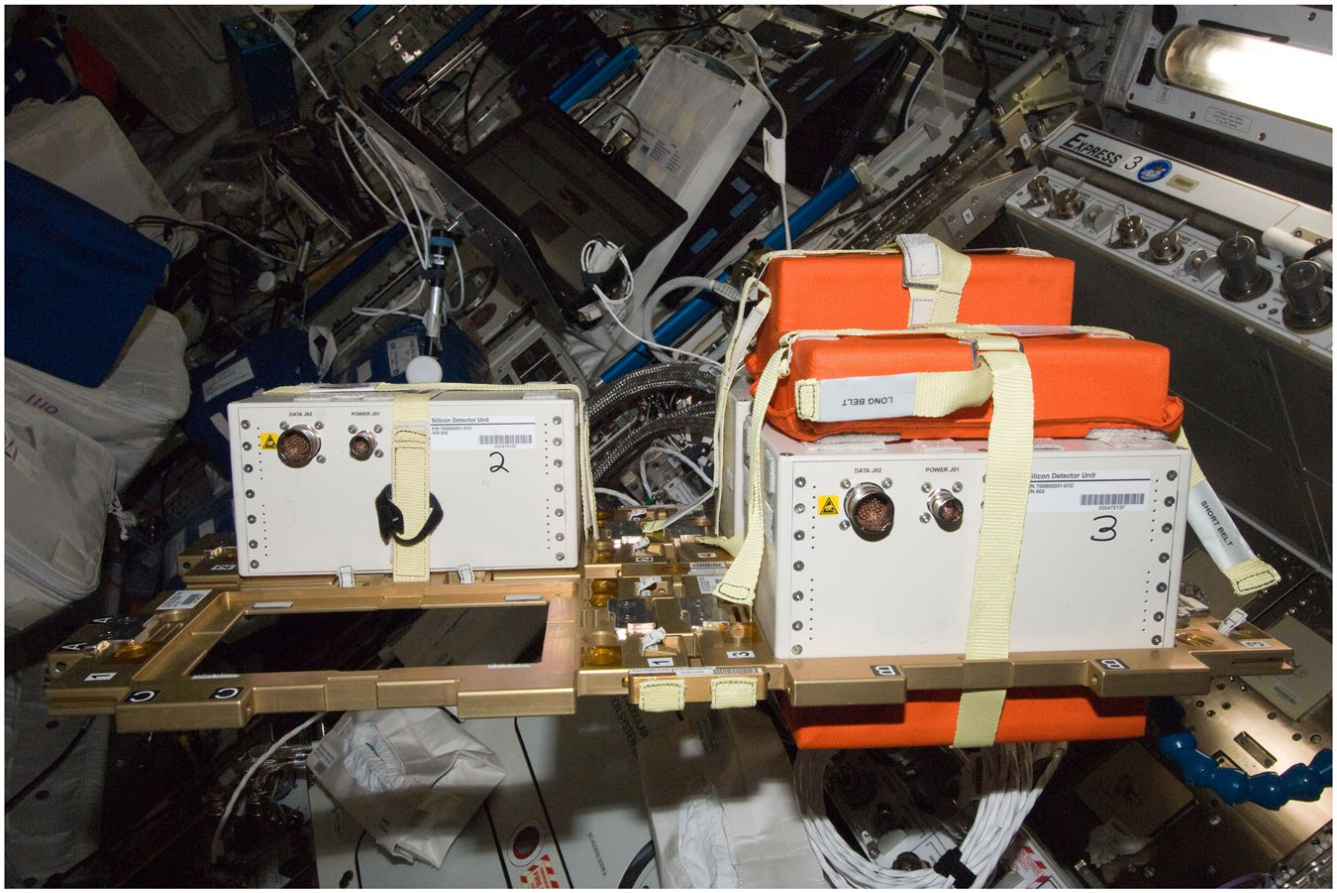
Configuration during the ALTEA-shield measurements. Three of the ALTEA SDUs are deployed on a flat configuration using a modular support structure that can hold four SDUs. The Digital Acquisition Unit (DAU) will be positioned in the empty space visible in Fig. 2. One SDU (n. 2, on the left) is the reference SDU, without the shielding tiles. Tiles of different thickness are positioned on the SDUs n. 1 and n. 3 (on the right, in orange).

The ALTEA-shield project is part of an ESA program aimed at using the ALTEA detector system^9–11^ to measure the radiation environment in the USLab module of the ISS and to investigate the shielding effectiveness of Kevlar, compared to the Polyethylene one. The shielding measurements have been carried out in the Columbus module of the ISS from June 8^th^ 2012 to November 13^th^ 2012. Table 1 lists the projects phases and Fig. 1 shows the experiment setup.

**Table 1:** Schedule of the ALTEA – shield project.

Date	Material	Total Duration (days)	Position
08-Jun-2012 00:00 08-Aug-2012 23:59	Polyethylene	62	ER3 Columbus
09-Aug-2012 18:09 30-Sep-2012 14:22	Kevlar	52	ER3 Columbus

Data taking periods are reported in the first column for the two materials listed in the second column. The total duration includes short ‘off’ periods due to many causes such as maintenance and transient electronic failures. The measurement site was inside the Express Rack 3 (ER3) in the Columbus module of the International Space Station. Kevlar measurement period ended on November 13^th^ 2012. We use only data up to September 30^th^ for electronic noise issues.

The ALTEA detector system is composed by six identical Silicon Detector Units (SDUs) and a Data Acquisition Unit (DAU). Each ALTEA SDU is a particle telescope with six silicon planes, able to determine the energy loss and the trajectory of passing-through cosmic-ray ions^7^.

The three SDUs are mounted on a modular support structure (Fig. 1) arranged in a flat configuration with the three SDUs facing the same direction.

**Figure 2:**
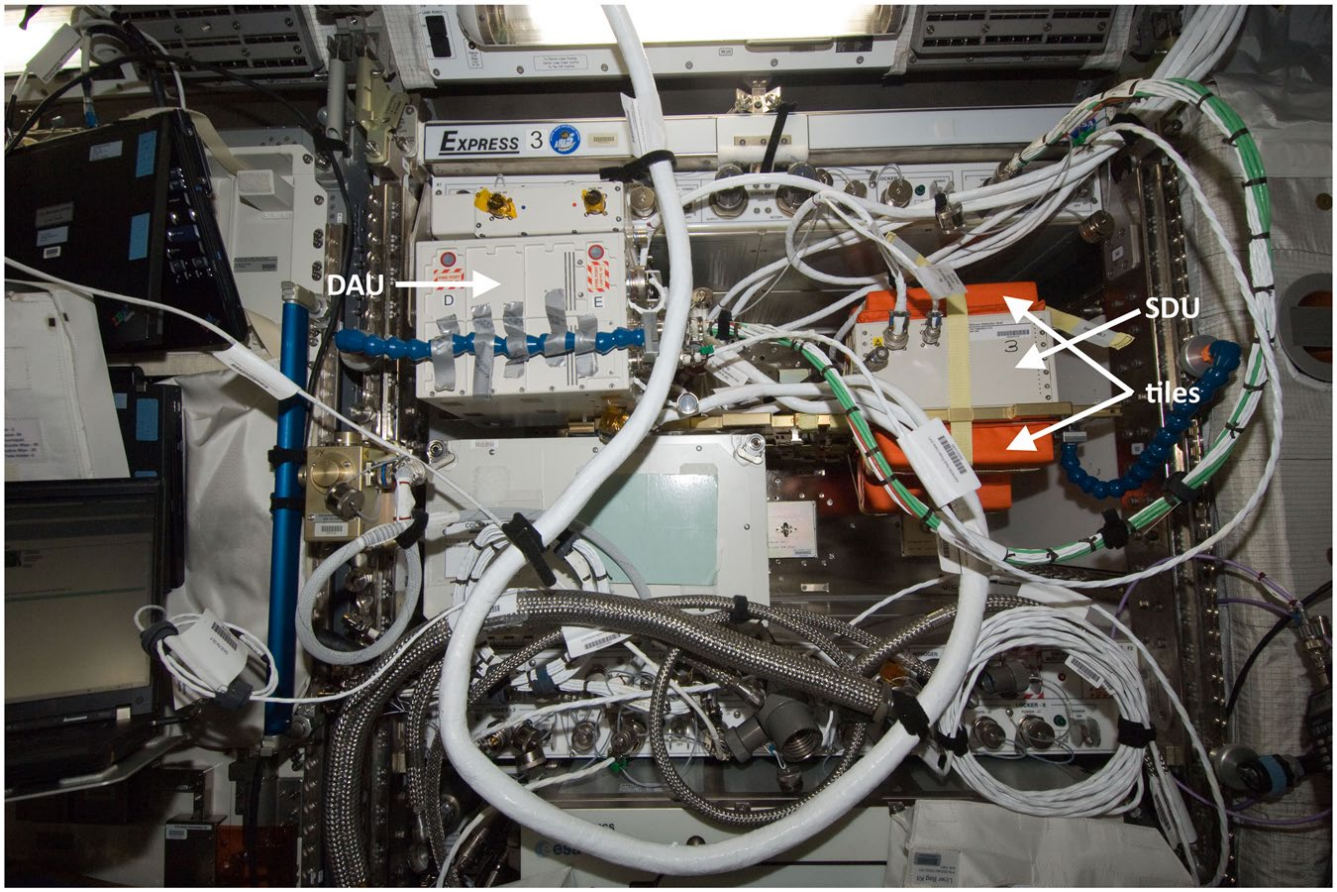
Position of the ALTEA-shield system. The ALTEA system is shown in its measurement location inserted in Express Rack 3 in the Columbus module.

The modular support structure can host 4 SDUs: the empty holding plate is used to host the DAU (Data Acquisition Unit). The whole system is inserted in a double drawer in the Express Rack 3 in the Columbus module (see Fig. 2). In this configuration the SDUs were oriented along the ISS Z axis (ISS coordinate: Nadir - Zenith), which is likely the least shielded one^2, 12^.

The presented results consider the data taken when the ISS was at High Latitude (*HL*), where the radiation environment best mimics the deep space one (see the Methods section).

**Figure 3:**
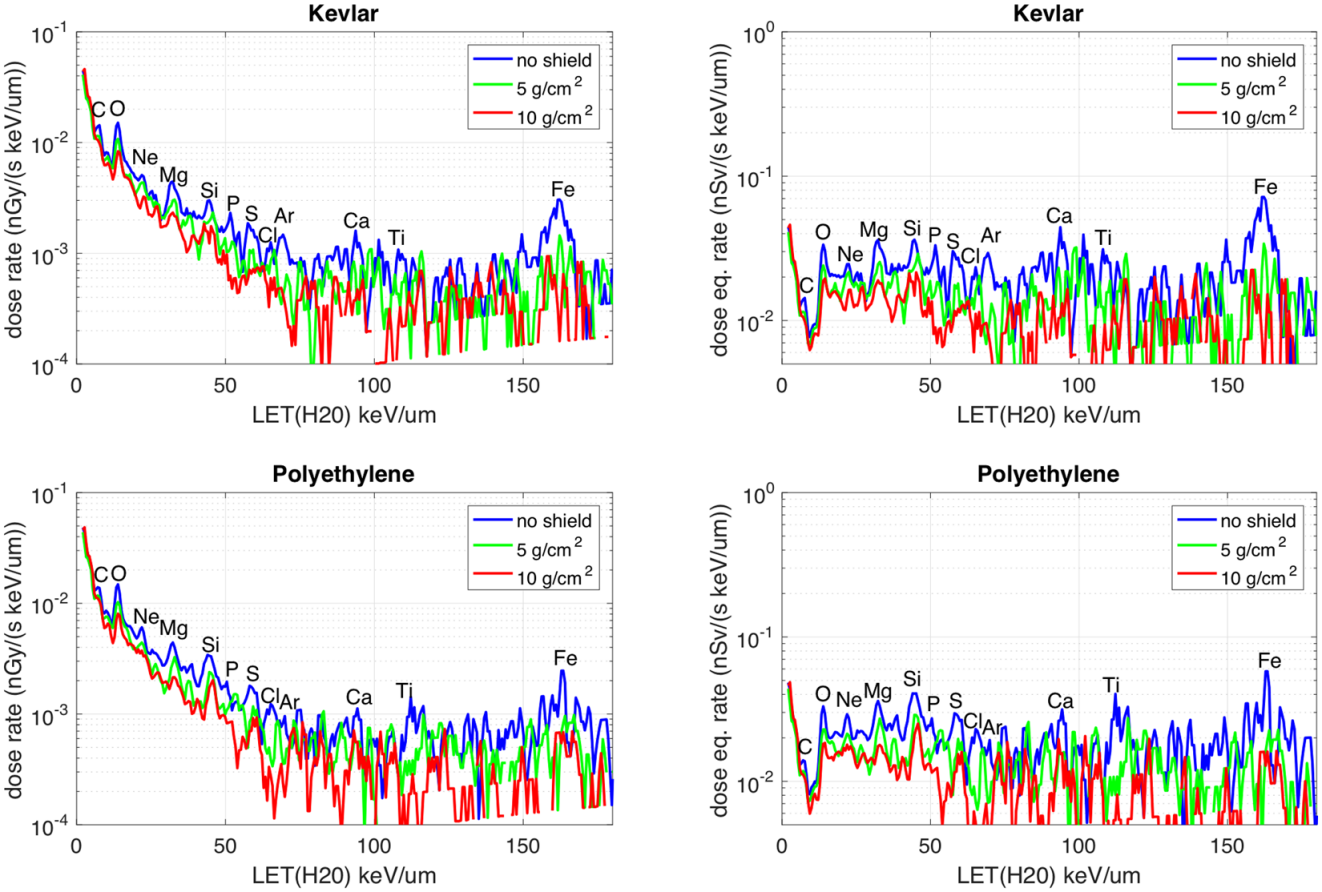
Dose rate and Dose equivalent rate spectra. Dose rate (left) and dose equivalent rate (right) spectra for Polyethylene (lower plots) and Kevlar (upper plots). Spectra are plotted vs. Linear Energy Transfer (LET, in water). Each spectrum shows three traces: blue = reference measurements with no shield; green = measurements with 5 g/cm^2^ shield tiles; red = measurements with 10 g/cm^2^ shield tiles. The symbols of the elements of the most prominent peaks are indicated.

Figure 3 shows the dose rate and dose equivalent rate spectra, for both Kevlar and Polyethylene. As described in the method section, the spectra are shown selecting ions passing in the detector within a Φ_2_ = 0.2 rad angle from the normal to the silicon planes. Due to this acceptance angle the distance travelled in the tile is equal to the material thickness, within 2%. All ions travel through the whole thickness of the smaller tiles (5 g/cm^2^). Part of the ions (≈10% for Polyethylene and ≈20% for Kevlar), enter or exit from the sides of the thickest tile (10 g/cm^2^), partly reducing the effectiveness of the 10 g/cm^2^ tile (see method section). With this angular selection the bi-directional geometrical factor of each SDU is 31 cm^2^ sr.

**Figure 4:**
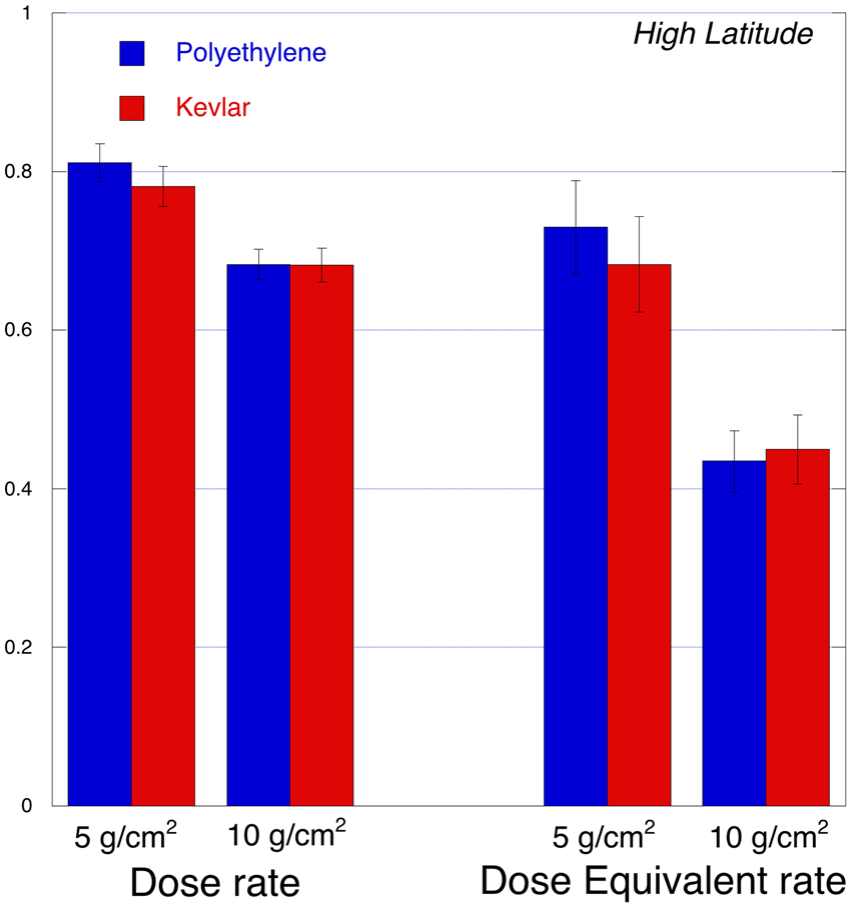
Dose rate and Dose equivalent rate ratios. Integrated Dose and Dose equivalent ratios (shielded measurements divided by not shielded measurements) for both Polyethylene (in blue) and Kevlar (in red); Ratio equal to one means that the shield doesn’t produce any reduction. Integration is performed in Linear Energy Transfer (LET) in water from 1.6 keV/µm to 207 keV/µm.

The spectra are then integrated, for a linear energy transfer (LET) in Silicon from 3 keV/µm to 350 keV/µm LET, corresponding to 1.6 keV/µm to 207 keV/µm in water. The results from the shielded spectra (green and red lines in Fig. 3) are divided by the results from the unshielded spectra (blue line), producing a bar plot for the radiation-shielding effectiveness, shown in Fig. 4. As described in the method section, in these integrated results an angular selection narrower than the previous one is performed (Φ_1_  = 0.11 rad), assuring that all ions travel through the full tile thickness with a path length equal to the tile thickness within 0.5%.

For an easier comparison with other works the results in Fig. 4 are presented as ‘percent of dose reduction’ in Table 2.

**Table 2:** Percent of dose reductions. Results are calculated from the difference between the unit and the ratios shown in Fig. 4.

		Polyethylene	Kevlar
Dose rate	5 g/cm^2^	19 ± 2	22 ± 3
	10 g/cm^2^	32 ± 2	32 ± 2
Dose equivalent rate	5 g/cm^2^	27 ± 6	32 ± 6
	10 g/cm^2^	57 ± 4	55 ± 4

**Figure 5:**
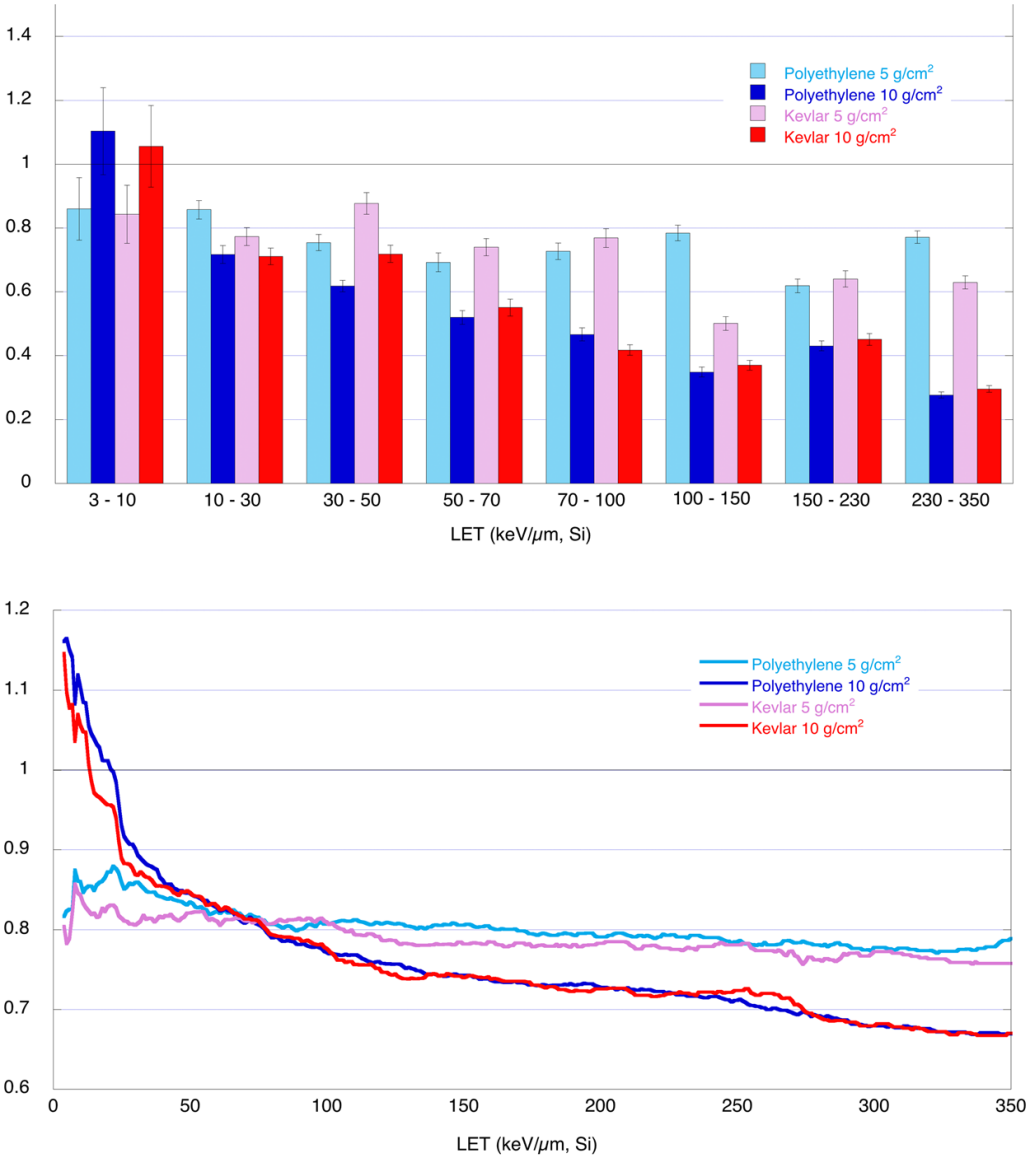
Dose rate ratios and cumulative ratios for different LET ranges. Top: as in Fig. 4 (left side), but in 8 different LET intervals of different width. Bottom: cumulative ratio (ratio of the cumulative dose rate for the shielded measurements to the cumulative dose rate for the unshielded measurements). LET in Silicon. Full colors: tiles with 10 g/cm^2^, patterned colors: 5 g/cm^2^; blue/cyan: Polyethylene, Red/magenta: Kevlar.

Figure 5 shows the evolution of the shielding effectiveness across LETs. In the top part, the ratio between the shielded (5 and 10 g/cm^2^) to unshielded dose rate measurements is presented. The population of LET bins is fast decreasing with LET (see Fig. 3), so the ratio of dose rate (shielded to unshielded) in this figure is integrated in LET bins of increasing width (from 7 keV/µm to 120 keV/µm). In the bottom part the cumulative ratio (calculated as the ratio of the shielded cumulative dose rate to the unshielded cumulative dose rate) is presented. Ratio values are presented versus LET in Silicon. The errors are evaluated from the single bin variance.

## Discussion

**Figure 6:**
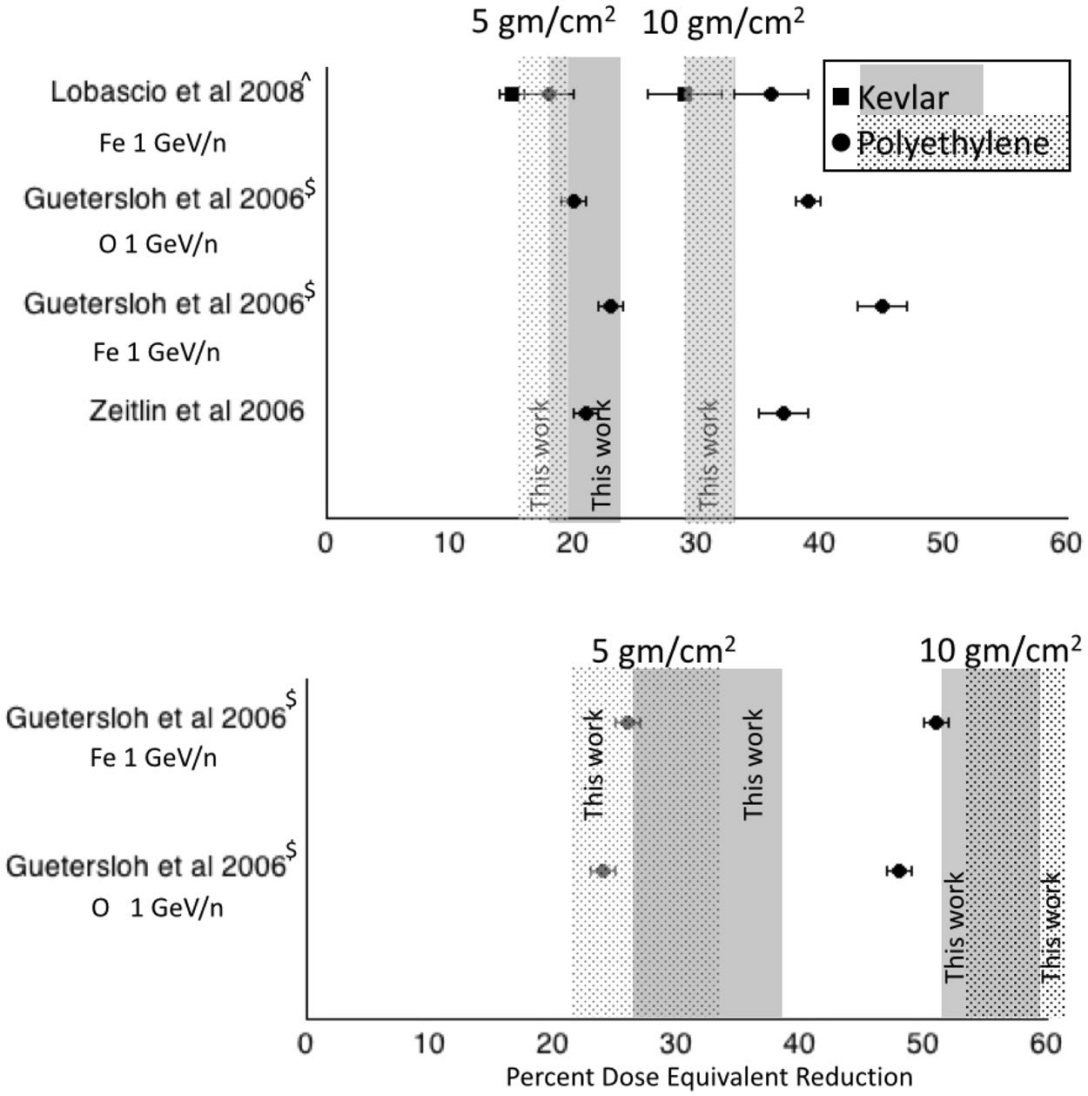
Comparison of our results (grey areas, thickness indicating ±σ) with other published results. Full gray area: our Kevlar results; patterned gray area: our Polyethylene results. Squared symbols: ground Kevlar results; round circles: ground Polyethylene results. Errors correspond to standard deviations (σ). Our two results for the dose at 10 g/cm^2^ are identical for the two materials. ^$^Extrapolated from values at 2.83 g/cm^2^, assuming linear relationship. In the paper other projectile species are considered. ^^^Extrapolated from values for unit areal density, assuming linear relationship.

The results of our measurement in space demonstrate that the Kevlar performances as radiation shielding material are as good as the Polyethylene ones. Specifically, for the 5 g/cm^2^ and the 10 g/cm^2^ thicknesses we report 22 ± 3% and 32 ± 2% for the dose rate reduction and 32 ± 6% and 55 ± 4% for dose equivalent rate reduction, respectively. These results are in agreement with previous ones from ground measurements (see Fig. 6) and are also supported by the similar chemical content of the two materials.

In ground measurements^5^ Polyethylene is reported to feature a dose reduction per unit areal density of 3.5 cm^2^/g and Kevlar 2.9 cm^2^/g. These results are obtained using 1 GeV/n Fe-ions. In another paper^3^ Polyethylene features 21% of dose reduction in 5 g/cm^2^ and about 37% for 10 g/cm^2^.

Another paper^4^, using 1 GeV/n ^16^O ions, shows about 18% of dose reduction for Polyethylene (5 cm) and 31% for 10 cm. In the same paper the dose reduction per unit areal density measured with a polyethylene shield of 2.83 g/cm^2^ clearly increases with projectile input energy, ranging in case of Fe-ions (0.6–1 GeV/n) from 2.9% to 4.5%, whereas, for example, for 0.6 GeV/n ^16^O there is 3% of dose reduction and 3.9% for 1 GeV/n. In general, the dose reduction per unit areal density is always less then 5.1%. The same data, for dose equivalent, range from 11% to 13.5% and from 8.4% to 14.6%. In general the dose equivalent reduction is less than 17.9%.

Figure 6 summarizes these results and compares them with the results obtained in the present work (shown as shadowed areas). There is no strong disagreement among the results. This is remarkable, considering the different approach followed: on ground single ions at a specific energy in one case, in space continuous spectra of ions in the other.

In the only reported measurements in space^6^, Kevlar tiles (5 g/cm^2^) on the ISS are shown to provide non-significant dose equivalent reduction when measured with Thermo-Luminescent Dosimeters (TLDs) passive detectors^13^. In the same paper relative abundances of N and O appears to increase when shielded by 5 g/cm^2^ Kevlar when measured using Alteino^14^, a detector similar to ALTEA. This should be compared with results in Fig. 3, specifically for the O peak, at about 13 keV/µm. Our results show that there is about 50% reduction of that peak. These discrepancies may be due to the different ways the measurements are carried on. In the mentioned work^6^, baseline and shielded measurements were not performed simultaneously and possible changes of the radiation environment may have occurred. Furthermore, in the Alteino measurements data from all the field of view are considered, including ions travelling mostly outside the tiles, reducing the measured shielding efficacy. Finally, the Alteino measurements considered data acquired along the entire orbit.

In several peaks (see for example the Si peak at 44 keV/µm), a reduction of the mean energy of the ions crossing the shield and leading to higher transferred energies, is observed. This results in an apparent shift of the transferred energy peak to the right.

The data in Fig. 5 support the evidence for a quite similar behavior of Kevlar and Polyethylene.

The enhancement of the 10 g/cm^2^ shown in the top bar plot (for both materials) is the reason for the higher values in the cumulative ratio. This effect is reversed only for LETs larger than about 70 keV/µm (bottom plot).

Two effects are likely the cause of this enhancement. The first and probably larger effect is the change of acceptance window of ALTEA. The tiles slow down the impinging ions, and the thicker tile produces the largest energy decrease. The ALTEA acceptance window for protons is about 25–45 MeV^7^ and it hardly measures GCR protons (due to the geomagnetic shielding most of the measured protons are albedo or secondary, see methods section). Interposing a shield would therefore lower the energy of the protons able to penetrate the Earth magnetic field at the ISS position, to a value possibly low enough to be measured by ALTEA. This protons contribution, therefore, add up to the albedo and secondaries ones. This effect is increasing the ratio values at low LET (in the first LET bin) for the 5 g/cm^2^ tile thickness and even more so for the thicker tiles. Unfortunately, an unknown and often changing amount of external shielding (ISS hull, racks, instrumentations, etc.) is interposed between ALTEA (plus tiles) and the external environment preventing to quantify this effect, even using simulations.

A second effect is the fragmentation of larger Z ions. This effect is mitigated by the single-track selection (see methods) where at most one fragment is taken into account for each impinging heavy ion and therefore the total number of fragments is at most the number of heavy ions (which is small). This effect should account for a small percentage of the measured enhancement.

Finally, an overall comment about the radiation shielding material research. As mentioned, the highly hydrogenated materials perform the best as shielding material in space^1^: liquid Hydrogen would therefore be the optimum choice, if it were safe. Kevlar shows performances as good as the Polyethylene ones, whose shielding effectiveness is lower than the liquid hydrogen one by only a factor 0.5. Therefore, not much room is left in terms of materials performances in space as passive radiation shielding. The future passive shielding research activity should therefore be not just towards “better materials”, but should aim at an integrated, synergic approach to the shielding issue. This approach would consider different passive elements, using materials with multi-purpose characteristics, starting from the habitat construction process, and possibly using active shielding^15, 16^ as well as pharmacological countermeasures. In this frame the excellent performance of Kevlar is not just due to its qualities as shielding material, successfully tested in this work, but more in general to its beneficial characteristics in many other areas, such as impact resistance, flexibility, etc., which makes it an optimal candidate as an element in the shielding integrated synergic approach mentioned above.

## Conclusions

This work describes the first measurement in space of material shielding efficacy using a concurrent measured baseline in a radiation environment similar to the deep space one. The material studied was Kevlar and its behavior has been compared to the one of Polyethylene, which is becoming a standard for passive radiation shielding in space. Our measurements show that the shielding effectiveness of Kevlar is comparable with the one of Polyethylene. For dose rate and dose equivalent rate, with 10 g/cm^2^ shielding material, a reduction factor better than 30% and 50% respectively, is documented.

Results are obtained considering high latitude data, where the deep space radiation environment is best mimicked. Two major features of these measurements (concurrent baseline and high latitude data selection) are essential for maximizing the measurement reliability.

Finally, Kevlar features of impact resistance and flexibility (also being a fabric) make it an optimal candidate as a performing element in an integrated shielding approach.

## Methods

### The ALTEA Silicon Detector Unit

Each of the six identical ALTEA SDUs is a particle telescope built with six parallel silicon planes^7^. Each silicon plane (380 µm thick) is composed by two squared silicon chips of 8 × 8 cm^2^, spaced by 5 mm, segmented in 32 strips with 2.5 mm pitch. Consecutive planes within an ALTEA SDU have orthogonal strip segmentation in order to reconstruct the x-y coordinate of the track, while the z direction is given by the relative position of the paired planes inside the SDU. The distance between two plane pairs is 37.5 mm. The structure results in a bidirectional Geometrical Factor (GF) of 230 cm^2^-sr per single SDU, calculated according to Sullivan^17^. The GF value has been also verified implementing a Monte Carlo code.

The Linear Energy Transfer (LET) range in silicon of the detector goes from a threshold of 2.9 keV/µm up to about 800 keV/µm. Each SDU is therefore able to measure protons from ≈25 MeV to ≈45 MeV, He from ≈25 MeV/n to ≈250 MeV/n and all other passing-through particles up to relativistic Molybdenum. The detector is triggered by passing-through particles that release more than 2.9 keV/µm on all the planes of an SDU^7^. The orbital information provided by the ISS is acquired off line and permits to study the distribution of particle fluxes into the three main geomagnetic zones^8^. In this paper we used the high latitudes (*HL*) selection (L > 3), where L is the McIlwain coefficient (L and the magnetic field B compose the ‘magnetic’ coordinates^18^).

### Tested materials

Kevlar, a fiber patented by Du Pont, is an organic fiber of the aromatic polyamide family. It features a unique combination of strength, toughness and thermal stability. Kevlar’s nominal density is 1.4 g/cm^3^. The selected fabric is Kevlar@29 Style 745 Resistant Fabric (Du Pont). Being a fabric, the tiles are manufactured sewing and overlapping several textile fabrics. This procedure leads to Kevlar tiles that have a density, which is about a factor of 2 lower than the nominal Kevlar fiber density (≈0.8 g/cm^2^ see Table 2).

Polyethylene (PE) is a synthetic resin, made from the polymerization of ethylene with a density ranging from 0.92 to 0.97 g/cm^3^. The selected PE is a high density (0.96 g/cm^3^) PE from Ensinger manufacturer (TECAFINE PE5).

During the measurements one of three SDUs is unshielded and used as reference. The other two are shielded with a different thickness of the same material (either Polyethylene or Kevlar). The tiles are placed on both views of each detector, as ALTEA cannot discriminate the entry-side of the passing-trough particle. One detector is shielded by tiles 5 g/cm^2^ thick, the other by tiles 10 g/cm^2^ thick, to investigate the relation between shielding efficiency and amount of shielding.

This data taking strategy, with simultaneous measurements with and without the shielding tiles, allows the measurement of the shielding effectiveness of the tile materials, ruling out the surrounding shield contribution. This technique also solves the problems due to the likely changing of material positions (moving racks, experiments, etc.) during the measurements.

Each tile covers an area 22 × 12 cm^2^, the different thicknesses are provided in Table 3.

**Table 3:** Thickness of the tiles.

Shield areal density (g/cm^2^)	Material	Thickness (cm)
5	PE	5
	Kevlar	6.5
10	PE	10
	Kevlar	13

The data are acquired in Real Time in the User Home Base in the University of Rome Tor Vergata, and stored in a Database for off line data analysis^19^.

### Data processing

The first step of data processing is pedestals (offset with no input) subtraction, performed for each silicon strip of the detector. Pedestal data are periodically acquired during the measurement by reading the detector response to an injected test charge. The second step is single track events selection. Each event is stored together with the relative geographical (latitude, longitude and height) and magnetic coordinates, for off-line selection of events occurring in a specific region.

### Angular selection

Not all ions that trigger an SDU travel through all the tile thickness (see also Fig. 1): some of them would exit (or enter) from the tile side. The use of larger tiles, to cover all the ALTEA field of view, would have been impractical. Furthermore, ion tracks normal to the tile surface have shorter path in the material than tracks at an angle. To minimize this phenomenon, we use the ALTEA tracking capability to evaluate the angle of the track, cutting at a narrow angle of incidence, in order to assume that all ions travel the same path-length in the material. This procedure decreases the geometrical factor and so the statistics. Even if this is acceptable for integrated calculations over all the energies, this solution heavily suppresses the statistics of the spectra. To present meaningful spectra we relax slightly the angular selection criteria, still obtaining a rather good selection of tracks travelling about the same length in the material. In particular, we used:

(i) a narrow angle range for integrated measurements. The analysis has been performed using data collected from ions impinging on the detectors within an angle Φ_1_ (with respect to the normal to the silicon planes, Φ = 0.11 rad = 6.3°). This selection ensures that the distance travelled in the material is almost constant and equal to the material thickness within the 0.5%. In this reduced acceptance configuration, the bidirectional geometrical factor of each SDU is 9.5 cm^2^-sr (correspondingly the efficiency of this angular selection is 4.1% of the full selection).
(ii) a narrow angle range for spectra. To improve statistics, the spectra have been calculated selecting a larger limit angle (Φ_2_ = 0.2 rad = 11°). The distance travelled in the materials, in this case, will be the material thickness within 2%. This larger acceptance further affects the measurements with 10 g/cm^2^ tiles (the thicker ones). In this case a few of the ions will travel partly outside the tiles (about the 10% for Polyethylene and about the 20% for Kevlar, because the Kevlar tile is thicker). When studying the spectra, this approximation for the 10 g/cm^2^ tiles should be considered. The bidirectional geometrical factor of each SDU for this angular selection is 31 cm^2^-sr (correspondingly the efficiency of this angular selection is 13.5% of the full selection).

**Figure 7:**
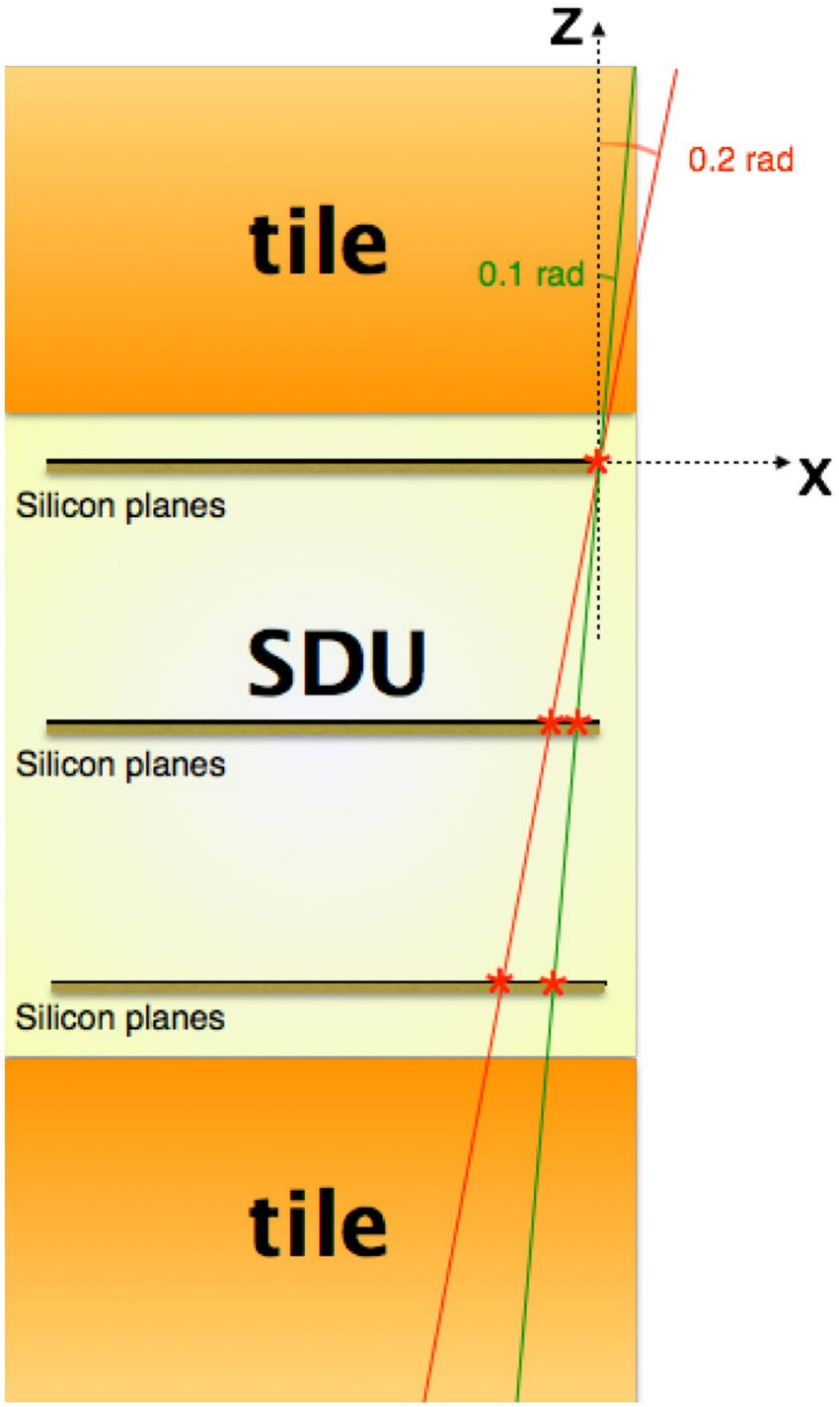
Schematic of the SDU with the two tiles on the top and on the bottom. The two drawn particle trajectories feature 0.1 rad (green) and 0.2 rad (red) inclination and are the most external ions with that inclination that can trigger the SDU (passing in all six silicon planes). The 0.2 rad most external trajectory passes only partly through the tile, while the 0.1 rad trajectory traverses all the tile.

In Fig. 7 a small graphic illustrates this geometrical issue.

In both cases the accepted direction is therefore mostly Z (nadir - zenith). Due to the geomagnetic shield, a GCR proton at the maximum latitude of the ISS must have at least about 150 MeV to reach the Station. Transported inside the ISS with an assumption of 6 cm of Al equivalent^20^ for the ISS shielding along Z axis, the proton would still have more than 60 MeV, well outside the proton acceptance window of ALTEA (see above). The real shielding can of course be different, however, this strongly suggests that the protons we are seeing are mostly albedo or secondary protons.

### Dose and Dose equivalent calculation

The measurement of the deposited Energy in Silicon (LET in Silicon) is converted to LET in water using the relation^21^:

1 $$\mathrm{Log}\,(LE{T}_{H2O})=-\,0.2902+1.025\,\mathrm{Log}\,(LE{T}_{Si})$$

And also

2 $$LE{T}_{k}{\rm{\Delta }}{l}_{k}={\rm{\Delta }}{E}_{k}$$

where *∆E*_*k*_ is the measured deposited energy by the particle *k*, and *∆l*_*k*_ is the path traveled by the particle *k* in the silicon plane. The selected narrow angles of incidence allow the approximation of *∆l*_*k*_ value with *h*, the thickness of the silicon planes, within ±0.5% for Φ_1_ or ±2% for Φ_2_:

3 $$LE{T}_{k}h \sim {\rm{\Delta }}{E}_{k}$$

The dose rate is:

4 $$Dose\,rate\,(\frac{nGy}{s}) \sim 1.6\frac{4\pi }{{\rho }_{{H}_{2}O}GF{\rm{\Delta }}T}\,\sum _{k=1}^{N}LE{T}_{k}$$

where *GF* is the geometrical factor (expressed in cm^2^ sr), *ρ*_*H2O*_ is the water density (in g/cm^3^), *∆T* (in s) is the acquisition time, *LET*_*k*_ (in keV/µm) is the measured *LET* of the *kth* particle and 1.6 is a numeric factor producing the Dose rate in nGy/s. The 4π coefficient comes from the integration over the full solid angle. The geometrical factor *GF* contains all the information about the geometry of the detector and it is used to estimate the radiation flux, assuming source isotropy.

To compute the dose equivalent rate we assigned different weights *w* to different LETs, according to ICRP^22^:

5 $$\begin{array}{ll}{\rm{LET}} < 10\,{\rm{keV}}/\mu {\rm{m}} & w=1\\ 10\,{\rm{keV}}/\mu {\rm{m}} < {\rm{LET}} < 100\,{\rm{keV}}/\mu {\rm{m}} & w=0.32\,{\rm{LET}}-2.2\\ {\rm{LET}} > 100\,{\rm{keV}}/\mu {\rm{m}} & w=300/{{\rm{LET}}}^{1/2}\end{array}$$

To construct the *LET* spectra, data is grouped in *M* bins (each 1 keV/µm, in silicon). In this case the dose rate can be calculated by summing over all the *LET* bins (*M*) the products of the average *LET* in each bin by the counts in the same bin:

6 $$Dose\,rate\,(\frac{nGy}{s})=1.6\,\frac{4\pi }{GF{\rm{\Delta }}T}\,\sum _{i=1}^{M}{C}_{i}LE{T}_{i}$$

where *C*_*i*_ is the counts in the i^th^ bin and it has been considered that ρ_*H2O*_ = 1 g/cm^3^.

### Errors evaluation

From (6) the error of the dose rate can be written as

7 $$Er{r}_{Doserate}=1.6\frac{4\pi }{GF{\rm{\Delta }}T}\,\sqrt{\sum _{i=1}^{M}{C}_{i}^{2}LE{T}_{i}^{2}[\frac{1}{{C}_{i}}+{(\frac{\delta LET}{LE{T}_{i}})}^{2}]}$$

Where the first addendum in the square bracket represents the statistical counting error and the second takes into account the uncertainties in the *LET* measurements. We assumed two *ADC* channels as uncertainty corresponding to 0.07 keV/µm in LET (flight calibration^11^).

#### For Dose equivalent each *LET*_*i*_ is replaced by *w*_*i*_*LET*_*i*_

Results are presented in a comparative way: data coming from the unshielded SDU are compared with the data concurrently acquired by the other two SDUs with tiles of different thicknesses. In this way any difference in impinging flux, due either to the external modulation or to the variation of the intervening structures inside the ISS, is taken into account.
